# Chemical Composition and Antioxidant Activity of Essential Oils from *Cinnamodendron dinisii* Schwacke and *Siparuna guianensis* Aublet

**DOI:** 10.3390/antiox2040384

**Published:** 2013-11-26

**Authors:** Milene Aparecida Andrade, Maria das Graças Cardoso, Juliana de Andrade, Lucilene Fernandes Silva, Maria Luisa Teixeira, Juliana Maria Valério Resende, Ana Cristina da Silva Figueiredo, José Gonçalves Barroso

**Affiliations:** 1 Department of Chemistry, University of Lavras, Lavras, MG 37200-000, Brazil; E-Mails: mileneaandrade@hotmail.com (M.A.A.); juandrade_quimica@yahoo.com.br (J.A.); lufernandes1000@hotmail.com (L.F.S.); teixeira_ml@hotmail.com (M.L.T.); 2 Department of Food Science, University of Lavras, Lavras, MG 37200-000, Brazil; E-Mail: jumvalerio82@hotmail.com; 3 Universidade de Lisboa, Faculdade de Ciências de Lisboa, DBV, Instituto de Biotecnologia e Bioengenharia, Centro de Biotecnologia Vegetal, C2, Campo Grande, 1749-016 Lisboa, Portugal; E-Mails: acsf@fc.ul.pt (A.C.S.F.); jgbarroso@fc.ul.pt (J.G.B.)

**Keywords:** volatile oils, chemical composition, free radicals

## Abstract

The objectives of this study were to chemically characterize and evaluate the antioxidant activity of essential oils *Cinnamodendron dinisii* Schwacke (pepper) and *Siparuna guianensis* Aublet (negramina). The essential oil was isolated by hydrodistillation using a Clevenger modified apparatus, and the identification and quantification of constituents, through GC/MS and GC-FID analysis. The antioxidant activity was evaluated using β-carotene/linoleic acid system and the DPPH radical sequestering method. In chromatographic analysis, the majority constituents found in the essential oil of *C. dinisii* were bicyclic monoterpenes, α-pinene (35.41%), β-pinene (17.81%), sabinene (12.01%) and sesquiterpene bicyclogermacrene (7.59%). In the essential oil of the fresh leaves of *Siparuna guianensis* Aublet, acyclic monoterpene, β-myrcene (13.14%), and sesquiterpenes, germacrene-D (8.68%) and bicyclogermacrene (16.71%) were identified. The antioxidant activity was low by the β-carotene/linoleic acid test and was not evidenced by the DPPH test, for both oils evaluated.

## 1. Introduction

The use of medicinal plants by humans dates back thousands of years due to their medicinal and nutritional properties. Many natural compounds extracted from plants have important biological activities. Among these compounds, we highlight the essential oils, which are increasingly attracting the attention of various segments of industry due to their multiple functions, especially antioxidant and antimicrobial activities.

Essential oils are defined by ISO as the products obtained from different parts of plants through distillation by steam distillation, hydro distillation and pressing the citrus fruit pericarp techniques. They are complex mixtures of volatile and lipophilic substances, formed by the secondary metabolism of plants characterized by a pleasant odor most of them presented [1,2].

Essential oils are marketed by various companies as raw material for various products with applications in perfumery, cosmetics, foods, and as adjuncts in medicines, among others. There are approximately 300 essential oils of commercial importance in the world. In the food industry, essential oils, besides imparting aroma and flavor to food, have important antioxidant activity, a property that further encourages its use [3].

In foods, lipid peroxidation is responsible for the development of unpleasant flavors and odors, making them unfit for consumption, and causes other changes that may affect the nutritional quality due to degradation of fat soluble vitamins and essential fatty acids, as well affecting the integrity and safety of food. In biological systems, lipid peroxidation on cell membrane unsaturated lipids causes membrane damage, disrupting the metabolite exchange mechanisms, and may cause cell death, becoming largely responsible for early aging and cardiovascular disease, cataracts, immune system decline and brain dysfunction, among others [4,5].

To prevent lipid peroxidation of foods, synthetic antioxidants commonly used in the industry are BHA, BHT, TBHQ and PG. However, studies assessing the toxicology of these compounds have demonstrated their carcinogenic potential in animals. Given the evidence of problems that can be caused by the consumption of synthetic antioxidants, research has emerged with the goal of finding natural products with antioxidant potential, which are an alternative to substitute the synthetic compounds or even promote an association between them, order to reduce their amount in food. One advantage of natural antioxidants versus the synthetic is that legislation regarding them is more flexible, but the natural antioxidants, in most cases, have lower antioxidant capacities than those of synthetic compounds [4,6].

The antioxidant activity of volatile oils, even very pronounced, can hardly be attributed to the components alone since due to the complex chemical composition of these natural compounds, which may contain molecules with different functional groups, the magnitude of the antioxidant activity shown by them can be related to the effect caused by the interaction of all constituents present in the essential oil, from those present in a greater proportion to those present in minor amounts. This interaction may produce a synergistic effect, when the interaction enhances the effect of the oil, or antagonistic, when the interaction negatively affects the antioxidant activity of the oil in study, which makes it very important to investigate the antioxidant properties of essential oils without considering only its major components [7].

The *Cinnamodendron dinisii* Schwacke species is a tree, 10–20 feet high, of the family Canellaceae, commonly known as “pepper” or “cure-all”, because the bark of the trunk, with a spicy flavor like true pepper, possesses medicinal properties and is slightly numbing [8,9].

The species *Siparuna guianensis* Aublet is used as a medical resource medicinal among the population of Mato Grosso, where the decoction of its leaves is used to combat sinusitis symptoms, fever, rheumatism, migraine, flu, body aches and “malina”. It is popularly known as “negramina” and belongs to the family Siparunaceae [10].

Given the growing importance of essential oils in the world market, their biological potential and existing species diversity as yet unexplored, further studies are needed that make their use viable. The objectives of this study were to chemically characterize and evaluate the antioxidant activity of essential oils of *C. dinisii* and *S. guianensis*.

## 2. Experimental Section

### 2.1. Plant Material

Leaves *Cinnamodendron dinisii* and *Siparuna guianensis* were collected in February 2011, in the morning, in the Medicinal Plant Garden of UFLA and on the UFLA campus respectively. The *C. dinisii* collection site has the following coordinates: −21°13'49.0476" latitude, −44°58'27.4764" longitude and 933 m of altitude, and the *S. guianensis* collection site: −21°13'41.9952" latitude, −44°58'9.0048" longitude and 951 m of altitude.

The species were duly identified and a voucher specimen of each species is registered in with the ESAL Herbarium, located in the Biology Department of UFLA, with the following registration numbers: 26,285 (*Cinnamodendron dinisii* Schwacke) and 26,623 (*Siparuna guianensis* Aublet).

The collected material was sent to the Laboratory of Organic Chemistry—Essential Oils of the Chemistry Department, Federal University of Lavras (UFLA) Lavras/MG, in which mature healthy leaves were selected with no injuries and blemishes caused by pathogens, insects or sunburn. The plant material was properly packaged and maintained under refrigeration (≈7 °C) until extraction of essential oils.

### 2.2. Essential Oil Extraction

The essential oil extractions were carried out in the Laboratory of Organic Chemistry—Essential Oils, Federal University of Lavras. The extraction method was hydro distillation using a modified Clevenger apparatus [11].

Three repetitions of the extraction from each plant were conducted using 300 g of fresh, chopped leaves for each repetition, distilling for 2 h. After this time, the oil was separated from the hydrolyte by centrifugation at 1100 g for 5 min, using a bench top centrifuge with horizontal crosspiece (Fanem Baby^®^ I Model 206 BL). The oil was removed with the aid of a Pasteur pipette, placed in a glass bottle, stored under refrigeration (4 °C), and protected from light [12].

### 2.3. Identification and Quantification of the Essential Oil Constituents

The identification of the constituents of the essential oils was performed by gas-liquid chromatography coupled to mass spectrometry (GC-MS), using an Autosystem XL equipped with a DB-1 fused silica column (30 m × 0.25 mm ID, film thickness 0.25 m; J & W Scientific Inc., Rancho Cordova, CA, USA) connected to a Perkin-Elmer Turbomass. The oven temperature was programmed from 45 to 175 °C in increments of 3 °C/min and subsequently at 15 °C/min to 300 °C. On reaching 300 °C, the temperature was kept isothermal for 10 min, temperature of the transfer line, 280 °C; temperature of the ionization chamber 220 °C, helium carrier gas, adjusted to a linear velocity of 30 cm/s; split-flow ratio 1:40, ionization energy 70 eV, ionization current, 60 μA; mass range, 40–300 u (atomic mass unit), scan time 1 s. The compounds were identified by comparison of their retention indices, relative to those of *n*-alkanes C_9_-C_21_ and by comparison with a library of mass spectra developed in the laboratory of the Centre for Plant Biotechnology, Faculdade de Ciências da Universidade de Lisboa—Portugal [13].

The content of each constituent was determined by gas chromatography (GC-FID) in a Perkin Elmer 8700 gas chromatograph equipped with two Flame Ionization Detectors (FID), a data processing system and an injector, in which two columns of different polarity were installed: DB-1 fused silica, with immobilized methyl silicone phase (30 m × 0.25 mm ID, film thickness 0.25 m; J & W Scientific Inc.) and DB-17HT fused silica (30 m × 0.25 mm ID, film thickness 0.25 mm; J & W Scientific Inc.). The oven temperature was programmed from 45 °C to 175 °C in increments of 3 °C/min and subsequently at 15 °C/min to 300 °C. On reaching 300 °C, the temperature was kept isothermal for 10 min. Injector and detector temperature, 290 °C and 280 °C, respectively. Hydrogen was used as carrier gas adjusted to a linear velocity of 30 cm/s. Split-flow ratio of 1:50. The percentage of oil constituents was determined by integration of peak areas without using correction factors. The values shown correspond to the average value of two injections [13].

### 2.4. Determination of Antioxidant Activity

#### 2.4.1. Reducing Method of Stable Radical DPPH

A DPPH methanolic solution was prepared at a concentration of 40 µg mL^−1^. For the evaluation, 2.7 mL of the stock DPPH solution were added in a test tube, followed by the addition of 0.3 mL of each dilution of the oil in methanol (300, 250, 200, 150, 100, 50; 25 g mL^−1^). In parallel, the control was prepared containing all reagents except the essential oil. After 60 min, readings were taken using a spectrophotometer (Shimadzu UV-160 1PC) at a wavelength of 517 nm [14].

The antioxidant activity was calculated as percentage inhibition of DPPH, using the following equation:

%*I* = 100 − (DPPHs/DPPHw/s)/100 (1)

where %*I* is the inhibition percentage of the DPPH radical, DPPHs corresponds to the absorbance of DPPH with the sample and DPPHw/s the absorbance of DPPH without sample (blank).

For comparison, activities of BHT, ascorbic acid and thymol standards were evaluated.

#### 2.4.2. Assay of β-Carotene/Linoleic Acid Oxidation System

In a round bottom flask, 60 µL of linoleic acid, 600 mg of Tween 20, 6 mg of β-carotene and 30 mL of chloroform were added. All the chloroform was removed using a rotary evaporator with a water bath at 50 °C (Büchi Rotavapor R 114). Subsequently, the residue was dissolved in 150 mL of distilled water saturated with oxygen under vigorous stirring. In test tubes, 2.7 mL of this solution were added to 0.3 mL of each oil (300, 250, 200, 150, 100, 50, 25 µg/mL), the control comprised only of methanol. The absorbance was measured immediately in a spectrophotometer (Shimadzu UV-160 1PC) at 470 nm [15].

After reading the initial absorbance, the tubes were incubated in a water bath at 50 °C for the oxidation reaction, a second reading being conducted after 60 min of incubation. All readings were performed in triplicate.

According to Wang *et al.* [16] the antioxidant activity was expressed as percentage of inhibition after 60 min of incubation using the following equation:

%AA = 100 × (Drc − Drs)/Drc
(2)

where %AA is the inhibition percentage of β-carotene degradation, Drc is the degradation ratio of the control [(ln(*a*/*b*)/60], Drs the degradation ratio in the presence of the sample [(ln(*a*/*b*)/60], “*a*” corresponds to absorbance at time zero and “*b*” corresponds to absorbance at 60 min.

For comparison, activities of BHT, ascorbic acid and thymol standards were evaluated.

#### 2.4.3. Statistical Analysis

For both tests, a completely randomized design (CRD) was used with seven concentrations and three replicates for each sample (or standard). The SISVAR [17] statistical program was used. Data were subjected to analysis of variance and the averages obtained were subjected to regression to 5% probability.

The adjusted equations were used to calculate the IC_50_ and graphs were plotted with the %*I* values of the DPPH assay, or %AA for the β-carotene/linoleic acid system versus the concentrations analyzed using the software Origin 6.0.

## 3. Results and Discussion

### 3.1. Identification and Quantification of the Constituents of Essential Oils

We identified 39 constituents present in the essential oil of *C. dinisii*, which are shown in Table 1. It can be observed that the essential oil presents almost entirely monoterpene hydrocarbons (76.20%), presenting as majority components bicyclic monoterpenes, α-pinene (35.41%), β-pinene (17.81%), sabinene (12.01%) and sesquiterpene bicyclogermacrene (7.59%). We noted the presence of a drimane-type sesquiterpene, drimenol (0.20%), present in the composition of many species of Canellaceae.

In the essential oil of the fresh leaves of *S. guianensis*, 41 constituents were identified (Table 1), which are composed mainly of sesquiterpene hydrocarbons (41.50%), oxygenated sesquiterpenes (19.40%) and monoterpene hydrocarbons (17.90%), respectively. As majority compounds, acyclic monoterpene, β-myrcene (13.14%) and sesquiterpenes germacrene-D (8.68%) and bicyclogermacrene (16.71%) were identified.

**Table 1:** Chemical composition of theessential oil of fresh leaves of *Cinnamodendron dinisii* and *Siparuna guianensis.*

		%*I* **	
RIc *	Compound	*C. dinisii*	*S. guianensis*
921	Tricyclene	t ***	t
924	α-Thujene	1.06	t
930	α-Pinene	35.41	1.83
938	Camphene	0.71	0.04
958	Sabinene	12.01	t
963	β-Pinene	17.81	0.86
975	β-Myrcene	1.46	13.14
995	α-Phellandrene	t	0.03
1000	δ-3-Carene	-	0.72
1002	α-Terpinene	0.24	-
1003	*p*-Cymene	1.21	t
1004	1,8-Cineole	4.37	-
1009	Limonene	1.54	1.23
1005	β-Phellandrene	t	0.06
1017	*cis*-β-Ocimene	1.99	0.03
1027	*trans*-β-Ocimene	1.82	t
1035	γ-Terpinene	0.75	-
1037	*trans*-Sabinene hydrate	0.15	-
1064	Terpinolene	0.17	t
1066	*cis*-Sabinene hydrate	0.15	-
1074	Linalool	0.65	-
1098	α-Campholenal	t	
1099	*trans*-*p*-2-Menthen-1-ol	0.09	-
1106	*trans*-Pinocarveol	0.11	-
1110	*allo*-Ocimene	0.03	-
1110	*cis-p-*2-Menthen-1-ol	t	
1114	*trans*-Verbenol	0.03	-
1121	Pinocarvone	t	
1134	Borneol	0.36	-
1148	Terpinen-4-ol	2.50	-
1153	Myrtenal	t	
1159	α-Terpineol	0.11	-
1168	Myrtenol	0.05	-
1265	Bornyl Acetate	0.10	t
1275	2-Undecanone	-	1.69
1332	δ-Elemene	0.32	0.58
1334	α-Terpinyl Acetate	0.32	-
1345	α-Cubene	-	0.04
1375	α-copaene	0.11	0.27
1379	β-Bourbonene	-	0.31
1388	β-Elemene	0.21	2.08
1385	β-Cubebene	-	0.18
1400	α-Gurjunene	-	t
1414	β-Caryophyllene	1.88	1.12
1426	γ-Elemene	-	0.05
1428	β-Copaene	-	0.04
1428	Aromandrene	0.23	0.04
1447	α-Humulene	t	2.07
1456	*allo*-Aromadendrene	-	0.05
1455	*trans*-β-Farnesene	0.20	-
1469	*trans*-Cadina-1(6)-4-diene	-	t
1474	Germacrene-D	-	8.68
1476	β-Selinene	t	0.20
1487	Bicyclogermacrene	7.59	16.71
-	Curzerene	-	2.15
1493	γ-Muurolene	-	t
1494	α-Muurolene	-	1.17
1500	(*trans*,*trans*) α-Farnesene	0.09	-
1500	γ-Cadinene	-	2.13
1505	*trans*-Calamene	-	0.29
1505	δ-Cadinene	0.14	1.04
1549	*trans*-Nerolidol	0.05	-
1533	Germacrene-B	-	2.34
1551	Spathulenol	1.88	4.16
1561	β-Caryophyllene Oxide	0.42	0.45
1566	Globulol	0.32	0.40
1569	Viridiflorol	0.16	3.00
-	Humulene epoxide II	-	0.63
1600	1-epi-Cubenol	-	0.15
1616	T-Cadinol	-	4.14
1620	β-Eudesmol	-	1.02
1626	α-Cadinol	-	1.95
1656	α-Bisabolol	-	3.53
1764	Drimenol	0.20	-
-	Atractylone	-	t
Total identified (%)		99.00	80.48
Monoterpene hydrocarbons		76.20	17.90
Oxygenated monoterpenes		9.00	0.00
sesquiterpene hydrocarbons		10.80	41.50
Oxygenated sesquiterpenes		3.00	19.40
Others		1.00	1.70

* RIc = retention index calculated; ** % = concentration in percentage; *** t = traces.

Some research was carried out to evaluate the chemical composition of essential oils obtained from different plant parts of the same species (or species belonging to the same family) evaluated in this study, as can be seen in Table 2.

**Table 2:** Chemical composition of the essential oils obtained from different plant parts of species of the Canellaceae family and of the *Siparuna guianensis* specie found by others authors.

Authors	Plant	Part of plant used	Place	Chemical composition (main compounds)
Torres *et al.* [18]	*Capsicodendron dinisii*	Bark	Guarapuava (PR)—Brazil	Monoterpenes—(68.5% of limonene).
Adams and Zanoni [19]	*Cinnamodendron ekamani*	Wood	Hispaniola—Caribbean island	1,8-cineole, α-humulene, β-caryophyllene, 4-terpineol, germacrene-D, β-elemene, α-pinene and α-terpineol.
Tucker *et al.* [20]	*Cinnamosma fragrans*	Commercial essential oil	Madagascar	1,8-cineole and sabinene.
Setzer [21]	*Canella winterana*	Leaves	Islands of Abaco—Bahamas	Myrcene, β-caryophyllene, *cis*- and *trans*-β-ocimene.
Amiguet *et al*. [22]	*Pleodendron costaricense*	Leaves and bark	Parrita (Costa Rica)	β-pinene, α-pinene, β-myrcene, β-thujene and β-caryophyllene.
Valentini *et al.* [23]	*Siparuna guianensis*	Leaves	Cerrado in Mato Grosso—Brazil	Sesquiterpenes and sesquiterpene hydrocarbon.
Antônio *et al*. [24]	*Siparuna guianensis*	Leaves	Panama	Curzerene, derivatives their degradation and myristicin.
Rebouças [25]	*Siparuna guianensis*	Leaves	Rio Branco (AC)—Brazil	γ-cadinene, bergamotene and β-caryophyllene.
Montanari [26]	*Siparuna guianensis*	Leaves	Tocantins (MG)—Brazil	α-terpinolene and α-bisabolol.
Fischer *et al.* [27]	*Siparuna guianensis*	Leaves and fruits	Brazilian cerrado	Leaves: decanoic acid and 2-undecanone. Fruit: undecanone-2, β-pinene and limonene.
Zoghbi *et al.* [28]	*Siparuna guianensis*	Leaves	Different places of the Amazon	Moju (PA): epi-α-bisabolol and spathulenol. Rio Branco (AC): spathulenol, selin-11-en-4α-ol, β-eudesmol and elemol. Belém (PA): germacrone, germacrene-D, bicyclogermacrene, germacrene-B and atractilone.

Torres *et al.* [18] evaluated the chemical composition of the volatile components present in the bark of *Capsicodendron dinisii* Schwancke, botanical synonym for *C. dinisii*, and verified the presence of 23 compounds (90% of the total oil composition), as 86.8% monoterpenes and limonene was found as the majority compound (68.5%). Similar results were observed in this study as to the class of compounds present in the highest percentage since 76.20% monoterpenes was found in the oil under study, but it was different regarding the majority component, limonene, that in the present work presented a percentage of 1.54%.

One can see how in the present study, the other studies described that evaluated the chemical composition of the essential oils of species present in four of the five genera of the family Canellaceae (*Cinnamodendron*, *Canella*, *Cinnamosma*, *Warburgia* and *Pleodendron*), presented, as a common characteristic, that all components are of terpenic origin, being mostly monoterpenes and sesquiterpenes.

Studies by Adams and Zanoni [19] evaluating the chemical composition of the essential oil obtained from wood of *Cinnamodendron ekamani*, a species of the same family as *C. dinisii*, verified the presence of 1,8-cineole (35.9%), α-humulene (9.1%), β-caryophyllene (6.5%), 4-terpineol (5.0%), germacrene-D (4.9%), β-elemene (4.8%), α-pinene (3.6%) and α-terpineol (3.0%). Subsequently Tucker *et al.* [20] observed the presence of 1,8-cineole (51.0%) and sabinene (10.6%) in the chemical composition of a commercial essential oil from Madagascar, named Mandravasarotra (*Cinnamosma fragrans* Baill., Canellaceae).

The essential oil of the leaves of *Canella winterana* (L.) Gaertn. (Canellaceae), from the islands of Abaco (Bahamas), presented a total of 19 compounds (100% of the total composition), the majority components being myrcene (32.4%), β-caryophyllene (18.8%) *cis*- and *trans*-β-ocimene (15.9% and 14.0%, respectively) [21]. Preliminary analysis of the essential oils of leaves and bark of *Pleodendron costaricense* indicated that the composition of the essential oils of the two parts of the plant analyzed were very similar, showing high levels of β-pinene, α-pinene, β-myrcene, β-thujene and β-caryophyllene with a lesser amount of linalool. The main difference between the volatile compositions of the two parts of the plant was the content of β-caryophyllene, which was the second most abundant component in the leaves, but almost absent in the bark [22].

The results obtained for the chemical composition of *S. guianensis* corroborate the results found by Valentini *et al.* [23], which identified and quantified the majority compounds of the volatile chemicals extracted from leaves of *S. guianensis* in an area of cerrado in Mato Grosso, for twelve months. They found that in relation to the variability of the classes of compounds in question, 70% of the identified compounds were sesquiterpenes and sesquiterpene hydrocarbons that appeared in the rainy season and dry-rainy season transition, with increased production in February 2008, the same month oil was obtained in the present study.

Antônio *et al*. [24] found, in the leaves of *S. guianensis* Panama, curzerene (25.64%), derivatives their degradation (42.31%) and myristicin (7.93%). In the same period, Rebouças [25] studied the essential oil of *S. guianensis*, plants collected in Rio Branco, Acre, and observed that the majority components of the essential oil were γ-cadinene (21.8%), bergamotene (14.2%) and β-caryophyllene (15.1%).

Subsequently, Montanari [26] noted that in the essential oil of plants collected in the city of Tocantins (MG), two constituents, the monoterpene α-terpinolene and the sesquiterpene alcohol α-bisabolol, together, accounted for about 80% of the oil composition throughout the year, results that differ from those found in our work where only the α-bisabolol was found, but in smaller quantities.

Essential oils from leaves and fruits of *S. guianensis* collected in the southeastern Brazilian cerrado presented as major constituents in the leaf oil, decanoic acid (46.6%) and 2-undecanone (31.7%), in the fruit oil mainly undecanone-2 (32.5%), β-pinene (19.6%) and limonene (13.6%) were found, results that differ from those found in this present study [27].

The essential oils of *S. guianensis* obtained from plants collected in different places of the Amazon showed differences in their constitution. In the essential oil sample collected in Moju (PA) were the majority constituents epi-α-bisabolol (25.1%) and spathulenol (15.7%), the essential oil sample collected in Rio Branco (AC) presented as majority compounds spathulenol (22.0%), selin-11-en-4α-ol (19.4%), β-eudesmol (10.0%) and elemol (10.0%), while in the oil sample collected Belém (PA) the presence was verified of germacrone (23.2%), germacrene-D (10.9%), bicyclogermacrene (8.6%), germacrene-B (8.0%) and atractilone (31.4%) as majority compounds [28]. The chemical composition of essential oils evaluated in our study showed similarities with the oil of the plant collected in Belém (PA) regarding the majority presence of germacrene-D and bicyclogermacrene.

Differences in content and chemical composition of essential oils extracted from a species often occur, since the production of secondary metabolites, including essential oils, is strongly influenced by the environment in which the producing organism is inserted, and the factors responsible for such variations are, seasonality, circadian rhythm, age and plant development, as well as the different plant organs, temperature, water availability, nutrients, altitude, atmospheric composition and attack of pathogens and herbivores [29,30]. 

### 3.2. Antioxidant Activity

By the β-carotene/linoleic acid method, the essential oils studied showed little antioxidant activity, but did not provide IC_50_ values (>300 μg mL^−1^) in the concentration ranges tested. Among standards tested, BHT (IC_50_ > 25 μg mL^−1^) was the most efficient, followed by thymol (IC_50_ 105.82 μg mL^−1^) and ascorbic acid (IC_50_ 118.15 μg mL^−1^), respectively (Table 3).

Employing the DPPH technique, antioxidant activity was not observed for the essential oils studied, but among the standards assessed, ascorbic acid (IC_50_ 44.36 μg mL^−1^) was more efficient than BHT (IC_50_ 48.84 μg mL^−1^) followed by thymol (IC_50_ > 300.00 μg mL^−1^) (Table 3).

**Table 3:** Antioxidant activity of essential oils of *S. guianensis* and *C. dinisii* and of the standards thymol, BHT and ascorbic acid by the β-carotene/linoleic acid test and the DPPH radical sequestration method.

Methods	β-carotene/linoleic acid	DPPH
Components	IC_50_ * (µg mL^−1^)	IC_50_ (µg mL^−1^)
*C. dinisii*	>300.00	NI **
*S. guianensis*	>300.00	NI
BHT	<25.00	48.84
Thymol	105.82	>300.00
Ascorbic acid	118.15	44.36

* IC_50_ = Inhibition Concentration of 50%; ** NI = no inhibition in the concentration ranges tested.

According to the results, it can be said that the essential oils rich in terpenes showed better values for the antioxidant activity in the β-carotene/linoleic acid system oxidation assay, as the β-carotene/linoleic acid method can be especially useful for investigations of lipophilic antioxidants and is appropriate for the investigation of antioxidant activity of essential oils. On the other hand, if polar compounds, such as ascorbic acid, were only tested by it, they would be considered weak antioxidants [31]. This explains the lower efficiency of ascorbic acid, compared with thymol.

According to Ruberto and Baratta [32], in lipid systems, phenolic compounds are effective antioxidants, thus, thymol and carvacrol molecules are indeed responsible for the antioxidant activity of many essential oils that contain them and a scant antioxidant activity is given to monoterpene and sesquiterpene hydrocarbons. Only three monocyclic components, terpinolene, α-terpinene and γ-terpinene, and to a lesser degree, sabinene (a bicyclic), show considerable activity. The presence of these strongly activated methylene group molecules, according to the authors, is probably the reason for this behavior. These results, may explain the low antioxidant activity of essential oils of *C. dinisii*, being composed of 87% of monoterpene and sesquiterpene hydrocarbons as well as *S. guianensis*, composed of 58.40% of this group of substances.

According to Mata *et al.* [33], the absence of antioxidant activity observed for the terpene compounds in the DPPH reduction can be explained by the fact that they are not capable of donating a hydrogen atom and the low solubility provided by them in the reaction medium of the assay, because this test utilizes methanol or ethanol as solvent. Thus, the fact that the essential oils of our study do not show significant antioxidant activity can be explained, since both oils are composed almost entirely of monoterpene and sesquiterpene hydrocarbons. For Viuda-Martos *et al.* [34], the cited factors can be considered as the main limitation of this assay for measuring the antioxidant activity of lipophilic samples, like many essential oils.

Despite the essential oils tested in this study not showing significant antioxidant activity, many essential oils have shown antioxidant potential. As an example there is the research conducted by Guimarães [35], who investigated the antioxidant activity of essential oils of *Lippia sidoides*, *Alomia fastigiata*, *Ocotea odorifera*, *Mikania glauca* and *Cordia verbenacea*, and their majority constituents, by the methods of the β-carotene/linoleic acid oxidation system, the formation of thiobarbituric acid reactive species (TBARS) and the reduction of the stable DPPH radical, and found that the essential oil of *L. sidoides* showed higher antioxidant activity, presenting the lowest IC_50_ values in all trials, and the antioxidant activity presented by the essential oil of *L. sidoides* was attributed to its majority constituent carvacrol, which also showed high antioxidant activity when assessed in isolation. It was also observed that there is influence of the methodology on the antioxidant activity presented by different essential oils and compounds evaluated, demonstrating the importance of the methodology for the determination of activity. Lima *et al.* [36] observed that the essential oils of *Myristica fragrans* and *Salvia microphylla* showed antioxidant potential by the β-carotene/linoleic acid test, with IC_50_ values of 976 and 770 µg mL^−1^, respectively.

## 4. Conclusions

We concluded that the main components found in the essential oil of the fresh leaves of *C. dinisii* were α-pinene (35.41%), β-pinene (17.81%), sabinene (12.1%) and bicyclogermacrene (7.59%) and the essential oil of the fresh leaves of *S. guianensis* were identified, β-myrcene (13.14%), germacrene-D (8.68%) and bicyclogermacrene (16.71%) and antioxidant activities of the essential oils were evaluated by testing low acid β-carotene/linoleic and were not evidenced by DPPH to test both oils evaluated.
